# Outbreak of CTX-M-15 Extended-Spectrum β-Lactamase-Producing *Klebsiella pneumoniae* ST394 in a French Intensive Care Unit Dedicated to COVID-19

**DOI:** 10.3390/pathogens10111426

**Published:** 2021-11-04

**Authors:** Cécile Emeraud, Samy Figueiredo, Rémy A. Bonnin, Mouna Khecharem, Souad Ouzani, Pierre-Etienne Leblanc, Agnès B. Jousset, Nicolas Fortineau, Jacques Duranteau, Laurent Dortet

**Affiliations:** 1 Department of Bacteriology-Hygiene, Bicêtre Hospital, Assistance Publique—Hôpitaux de Paris, 94270 Le Kremlin-Bicêtre, France; cecile.emeraud@aphp.fr (C.E.); mouna.kecharem@aphp.fr (M.K.); souad.ouzani@aphp.fr (S.O.); agnes.jousset@aphp.fr (A.B.J.); nicolas.fortineau@aphp.fr (N.F.); 2 UMR-S 1184, Paris-Saclay University, 94270 Le Kremlin-Bicêtre, France; samy.figueiredo@aphp.fr (S.F.); remy.bonnin@u-psud.fr (R.A.B.); 3 French National Reference Center for Antibiotic Resistance, 94270 Le Kremlin-Bicêtre, France; 4 Faculty of Medicine, Paris-Saclay University, 94270 Le Kremlin-Bicêtre, France; jacques.duranteau@aphp.fr; 5 Chirurgical ICU, Bicêtre Hospital, Assistance Publique—Hôpitaux de Paris, 94270 Le Kremlin-Bicêtre, France; pierre-etienne.leblanc@aphp.fr

**Keywords:** CTX-M-15, ESBL, *Klebsiella pneumoniae*, antibiotic resistance, hand-hygiene

## Abstract

Infections caused by extended-spectrum β-lactamase-producing *Klebsiella pneumoniae* (ESBL-KP) are constantly rising worldwide and are often reported as causative agent of outbreaks in intensive care units (ICUs). During the first wave of the COVID-19 pandemic, bacterial cross-transmission was thought unlikely to occur due to the reinforcement of hygiene measures and prevention control. However, we report here an ESBL-producing *K. pneumoniae* (ST394) isolate responsible for a nosocomial outbreak in an ICU dedicated to COVID-19 patients.

## 1. Introduction

*Klebsiella pneumoniae* is an important bacterial pathogen responsible for various infections, including nosocomial infections in intensive care units (ICUs). In addition, this bacterial species is a major cause of multidrug-resistant infections worldwide through the production of extended-spectrum β-lactamases (ESBLs) and carbapenemases [1,2].

ESBLs are Ambler class A enzymes able to hydrolyze third-generation cephalosporins and aztreonam but spare cephamycins (cefoxitin). These enzymes are inhibited by classical β-lactamase inhibitors such as clavulanate, sulbactam, and tazobactam [3], but also by novel β-lactamase inhibitors such diazabicyclooctane derivatives (e.g., avibactam and relebactam) and boronic acid derivatives (e.g., vaborbactam) [4].

Until the late 1990s, ESBLs produced by *K. pneumoniae* isolates were almost exclusively TEM and SHV enzyme variants [5]. Then, CTX-M enzymes (i.e., ‘active on CefoTaXime, first isolated in Munich’) replaced TEM and SHV mutants as the predominant ESBLs in many European countries [6]. In the 2000s, CTX-M enzymes became the most prevalent ESBL both in healthcare and community settings. Based on their amino acid sequences, CTX-M enzymes could be clustered into five main groups: CTX-M-1-, CTX-M-2-, CTX-M-8-, CTX-M-9-, and CTX-M-25-group [1]. CTX-M-1-type and CTX-M-2-type ESBLs were first related to chromosome encoded β-lactamase present in *Kluyvera ascorbata* [7], whereas CTX-M-8- and CTX-M-9-type enzymes are closer to the chromosome encoded β-lactamase of *Kluyvera georgiana* [8]. According to the BLDB database (http://www.bldb.eu/, accessed on 3 November 2021) 246 variants of CTX-M enzymes have been reported [9]. Initially, CTX-M enzymes were mainly found in *Escherichia coli* strains [10,11]. Now, the dissemination of these enzymes concerns all Enterobacterales, including *K. pneumoniae* [12]. Currently, CTX-M-15 is the most widely distributed CTX-M enzyme worldwide [6,13,14,15,16]. This variant belongs to the CTX-M-1 group. It differs from the CTX-M-3-type by a single amino acid substitution at position 240 (D240G) conferring an increasingly catalytic activity towards ceftazidime [17].

Several outbreaks of CTX-M-15-producing *K. pneumoniae* have already been reported in European countries such as France [18], Hungary [19], and Russia [20]. These studies, which investigated nosocomial outbreaks caused by a CTX-M-15-producing *K. pneumoniae* isolate, reported that *bla*_CTX-M-15_ was carried on different plasmids. In France, *bla*_CTX-M-15_ was found to be localized on a 160-kb plasmid that also carried *bla*_OXA-1_, *bla*_TEM-1_, and *aac*6’*-Ib-cr* genes [18]. This association between *bla*_CTX-M-15_, *bla*_OXA-1_, and *aac*6’*-Ib-cr* genes has also been observed in other outbreaks [19,21].

In March 2020, a huge increase in the number of patients hospitalized in ICUs was observed in many European countries due to the spread of SARS-CoV-2. In France, specific wards dedicated to the management of COVID patients have been implemented in many hospitals. In these wards, infection control and hygiene measures were drastically upgraded (i.e., contact and respiratory precautions) to protect healthcare workers. In this context, the risk of emergence of a nosocomial outbreak was thought to be at its lowest level. However, we report here a large outbreak of CTX-M-15-producing *Klebsiella pneumoniae* in an ICU dedicated to COVID-19 infected patients between March and June 2020.

## 2. Results

### 2.1. Description of the CTX-M-Producing K. pneumoniae Related Outbreak

**Figure 1:**
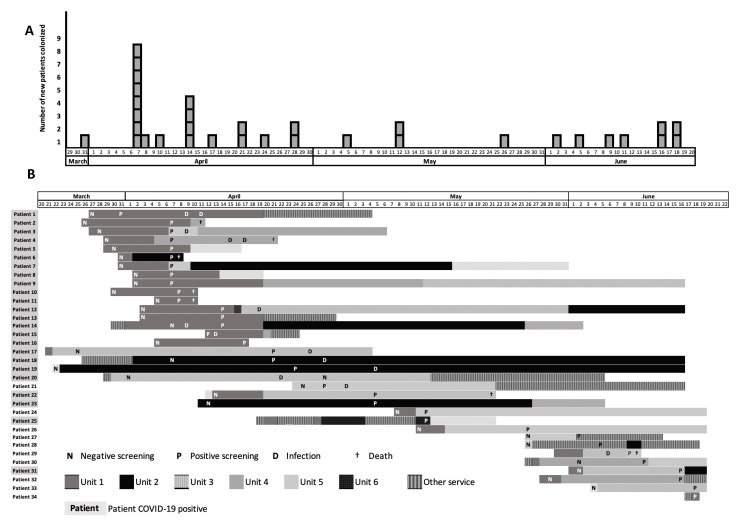
Timeline of the outbreak caused by CTX-M-15-producing *Klebsiella pneumoniae* in the different parts of an ICU in a French Hospital. (**A**) Time flow chart of CTX-M-15-producing *Klebsiella pneumoniae* recovered from 30 March and 20 June 2020, each square corresponds to one isolate. (**B**) Description of the stays of the different patient harboring the CTX-M-15-producing *Klebsiella pneumoniae* in the units of the ICUs from 20 March and 22 June 2020.

Between 30 March and 20 June 2020, 101 patients have been admitted to the surgical ICU of Bicêtre Hospital (37 for severe COVID-19 and 64 for other reasons) and 34 patients were colonized and/or infected with a CTX-M-producing *K. pneumoniae* isolate (Figure 1A). Temporal and geographic links were established between all patients (Figure 1B). Among the 34 patients (including 24 patients COVID-19 positive), 18 patients (52.2%) were colonized only, but 16 (47.1%) were also infected (9 patients with sepsis, 4 with pneumonia, one with urinary tract infection, one with meningitidis, and one with surgical site infection). The origin of the nine sepsis was a pneumonia, a urinary tract infection, and a catheter-related infection for three, two, and one patients, respectively, and was unknown for three patients. Eight of the 34 patients died during their ICU stay.

### 2.2. Bacterial Characterization and Clonal Relationship

Between 30 March and 20 June 2020, 83/515 rectal swab screenings were positive for ESBL-producing *K. pneumoniae* (from 34 patients). For all these strains, the NG-test CTX-M MULTI tests was positive, revealing the production of a CTX-M-type ESBL. All these strains possessed an identical resistance profile, with a resistance to third- and fourth-generation cephalosporins, aztreonam, piperacillin/tazobactam, amoxicillin/clavulanate, gentamicin, tobramycin, all fluoroquinolones, and co-trimoxazole according to CASFM (Figure S1). These strains remained susceptible to carbapenems, cefoxitin, temocillin, and amikacin.

Whole-genome sequencing was performed on all 34 CTX-M-producing *K. pneumoniae* isolates (one strain per patient). A unique resistome was identified for all strains, with the presence of several genes encoding resistance to β-lactams (*bla*_CTX-M-15_, *bla*_TEM-1B_, and *bla*_OXA-1_), aminoglycosides (*aac*(*3*)*-IIa-like*, *aac*(*6*′)*Ib-cr*), quinolones (*aac*(*6*′)*Ib-cr,* trimethoprime (*dfrA14-like*), tetracycline (*tetA)* and phenicols (*catB3-like*).

**Figure 2:**
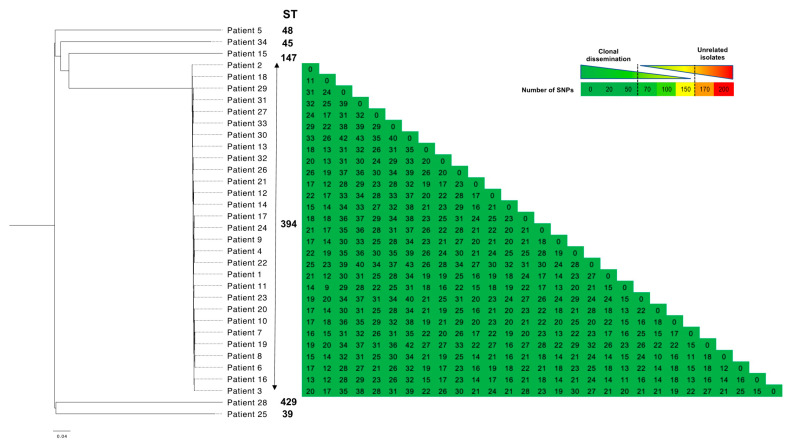
Phylogenetic tree with SNPs matrix (Single-Nucleotide Polymorphism) for the 22 CTX-M-15-producing *Klebsiella pneumoniae* sequenced in the study.

MLST analysis identified 29 strains belonging to the ST-394, and one to each ST-39, -45, -48, -147, and -429. On all 29 genomes of CTX-M-producing *K. pneumoniae* belonging to the ST-394, we used a core genome SNP-based (single-nucleotide polymorphism) approach to create a phylogenetic tree using the *K. pneumoniae* of the patient 1 as reference. Two strains were considered to be clonally related if they were separated by fewer than 50 SNPs along their common genome. The maximum number of SNPs observed between two strains was 43, confirming the cross-transmission of as a single strain (Figure 2).

### 2.3. Virulence

Despite the ST-394 CTX-M-15 producing *K. pneumoniae* isolate resulted in mucoid colonies, the string test was negative for all 34 strains. In addition, none of the seven virulence factor genes associated with hypermucous *Klebsiella* (*ybtS*, *mrkD*, *entB*, *rmpA*, *kfu*, *allS*, and *iutA*) were found in the genome. Within the ST-394 CTX-M-15 producing *K. pneumoniae*, two operons involved in iron uptake were present: the *ent*, *fep*, and *mrk* operons. However, these operons are found in other ST of *Klebsiella* that were not particularly reported as hypervirulent.

## 3. Discussion

ESBL-producing *K. pneumoniae* is mainly transmitted from patient to patient directly by the medical staff’s hands, or indirectly via the environment [22]. To prevent transmission, infection control measures, such as additional contact precautions and patient screening, must be implemented. In our study, we have highlighted the dissemination of a ST-394 CTX-M-15-producing *K. pneumoniae* in the ICU during the COVID-19 pandemic in France. Between 30 March and 20 June 2020, 29 patients were colonized and/or infected with this strain in the ICU. However, strains belonging to other STs (*n* = 5) did not disseminate in the ICU. Among the 29 ST-394 CTX-M-15 producing *K. pneumoniae* isolates, 16 (55.2%) were infected (including nine patients with sepsis) and no strain belonging to other STs caused infection. Geographic and temporal links were established between these patients. In addition, whole-genome sequencing analysis confirmed the dissemination of a unique strain of *K. pneumoniae* belonging to the ST-394. This particular ST was not described in any medical publication as being associated with ESBLs or being particularly virulent. In Europe, *K. pneumoniae* clinical isolates that produce CTX-M-15 are particularly found in the well-known “high-risk clones” of ST-307, ST-147, or ST-101 [23,24]. Several observations made in the ICU highlighted contributing factors linked to the pandemic context that can explain this massive dissemination of CTX-M-producing *K. pneumoniae*: surge of critically ill patients with a huge subsequent increase in workload, tight physical spaces and close proximity of patients in the common PAECU, increased pressure exerted on the healthcare system: recruitment of healthcare workers from other hospital services (less familiar with infection control measures), multiple medical and non-medical staff in contact with each patient, adoption of new safety measures such as the use of PPE (designed for self-protection, but also allowing the transmission of micro-organisms between patients as it could create a false sense of security among some users), and concomitant shortage of long-sleeved water-resistant gowns and hydroalcoholic solutions. Various measures were taken to stop this epidemic situation: (i) the reinforcement of gowns and hydroalcoholic solutions stock, (ii) the bleaching of siphons and sinks, and (iii) the education of medical and non-medical staff. The dissemination of this strain stopped quickly after the implementation of these measures. As previously reported in most of *bla*_CTX-M-15_-carrying plasmids, *bla*_OXA-1_, *bla*_TEM-1_, and *aac*6’*-Ib-cr* genes were associated with *bla*_CTX-M-15_ in this ST-394 *K. pneumoniae* isolate [18,19,21,25]. Usually, only 6% to 7% of the patients colonized with ESBL-producing *K. pneumoniae* further develop an infection with the same strain [26]. Here, 16/34 (47.1%) patients were further infected with this carriage ST-394 CTX-M-15-producing *K. pneumoniae* isolate. This particularly high prevalence (7-fold more than expected) of infection among colonized patients led us to look for specific virulence factors. The most studied hypervirulent *Klebsiella pneumoniae* are known to possess at least few genes among *ybtS*, *mrkD*, *entB*, *rmpA*, *kfu*, *allS*, and *iutA* [27]. In addition, these hypervirulent clones are reported to form hypermucoid colonies that lead to a positive string test. Here, the string test remained negative, despite hypermucoid colonies. In addition, the genome analysis did not identify any particular virulence factor encoding gene among *ybtS*, *mrkD*, *entB*, *rmpA*, *kfu*, *allS*, and *iutA*. Of note, the operons *ent* and *fep* were found. The *ent* and *fep* operons encode enterobactin and ferrienterobactin, respectively. These two siderophores were reported to be strongly implicated in bacterial virulence [28]. Moreover, the *mrk* operon was also identified in this strain. This operon, and more precisely *mrkCDF* encoding anchoring for type II fimbriae, was previously reported to be involved in transmigration of the bacteria through the intestinal epithelium [29]. However, these virulence factors are present in most of the *K. pneumoniae* isolates including ST that are not particularly hypervirulent. Accordingly, further experiments should be performed to link the potential implication of these operons with the high incidence of bacteremia that occurred in patient firstly colonized with this ST-394 *K. pneumoniae*

## 4. Materials and Methods

### 4.1. Context

The ICU is a 35-bed service located in a large teaching hospital in a suburb of Paris in France, with a total of 953 beds. This surgical ICU is usually divided into six different parts located on the same floor: four critical care units (CCU) with five single rooms in each one, one intermediate-care unit (IMCU) with eight single rooms and one common seven-bed post anesthetic and emergency care unit (PAECU; admission and intensive care for in-hospital and out-of-hospital vital emergencies and high-risk surgical patients). All these units share the same hospital staff. All patients traditionally undergo rectal swab screening for ESBL-producing Enterobacterales at the admission into the ICU and then once a week. During the first wave of COVID-19 pandemic in France (March–June 2020), the entire ICU (except the eight ACU beds) has been dedicated to patients with severe COVID-19 and the capacity of the common PAECU was increased from seven to eighteen beds (by transiently transforming 11 non-ICU PAECU beds into ICU beds). Institutional protocols on personal protective equipment (PPE) donning and doffing were distributed to all healthcare providers, leading to the upgrade of infection control and hygiene measures including contact and respiratory precautions. From 30 March to 20 June 2020, a rise in the number of patients colonized or infected with ESBL-producing *K. pneumoniae* was observed.

### 4.2. Screening, Bacterial Isolates, and Susceptibility Testing

The screening for ESBL-producing Enterobacterales in fecal samples was performed by plating rectal swabs on a chromogenic and selective agar: ChromID^®^ ESBL (bioMérieux, Marcy-l’Etoile, France). For all Enterobacterales cultured on this screening medium, the bacterial identification was performed via MALDI-TOF mass spectrometry (Microflex, Bruker Daltonics, Bremen, Germany) and antimicrobial susceptibility testing was performed by disc diffusion according to EUCAST guidelines. An immunochromatographic assay NG-test CTX-M MULTI (NG-Biotech, Guipry, France) [30] was performed on all ESBL-producing *K. pneumoniae* isolates.

### 4.3. Bacterial Characterization and Clonal Relationship

Whole-genome sequencing was performed on 34 CTX-M-producing *K. pneumoniae* isolates using the HiSeq system (Illumina Inc, San Diego, USA). A single strain of CTX-M-producing *K. pneumoniae* was sequenced per patient. For each strain, the acquired antimicrobial resistance genes were identified using Resfinder 4.1 (https://cge.cbs.dtu.dk/services/ResFinder/, accessed on 3 November 2021) and MultiLocus Sequence Typing (MSLT) was performed using the MLST 2.0 server (https://cge.cbs.dtu.dk/services/MLST/, accessed on 3 November 2021).

Single nucleotide polymorphism (SNP) analysis was performed on 34 whole genomes using the CSIphylogeny V1.4 server (www.cge.cbs.dtu.dk/services/CSIPhylogeny/, accessed on 3 November 2021) with parameters as follows: select min depth at SNP position at 10X, minimum distance between SNPs at 10 bp, and minimum SNP quality at 30. Phylogeny was performed using the CSIphylogeny v1.4 server and visualised using FigTree software v1.4.3 (http://tree.bio.ed.ac.uk/software/figtree/, accessed on 3 November 2021). A SNP matrix was built from data obtained on the CSIphylogeny, in order to compare the CTX-M-producing *K. pneumoniae* isolates.

### 4.4. Virulence

The high incidence of bacteremia in patients carrying this particular CTX-M-producing *K. pneumoniae* isolate (ST-394) led us to look for virulence factors. First, a string test was performed to identify a hypermucous phenotype. Then, whole genomes of the 34 strains were analyzed to identify the seven genes of virulence factors known to be expressed by known hypervirulent *Klebsiella pneumoniae* (*ybtS*, *mrkD*, *entB*, *rmpA*, *kfu*, *allS*, and *iutA*) [27].

## Data Availability

Data is contained within the article or supplementary material.

## Supplementary Materials

The following are available online at https://www.mdpi.com/article/10.3390/pathogens10111426/s1, Figure S1: Antibiogram of the CTX-M-15-producing Klebsiella pneumoniae responsible of the outbreak.
