# Supplementary material for: Outbreak of CTX-M-15 Extended-Spectrum β-Lactamase-Producing Klebsiella pneumoniae ST394 in a French Intensive Care Unit Dedicated to COVID-19
Source: Pathogens. 2021 Nov 4;10(11):1426. doi: 10.3390/pathogens10111426 (PMC8618658; doi:10.3390/pathogens10111426)
Supplement: Supplementary file 1 [file pathogens-10-01426-s001.zip › pathogens-1434410-supplementary.pdf]

Figure S1. Antibiogram of the CTX-M-15-producing *Klebsiella pneumoniae* responsible for the outbreak.

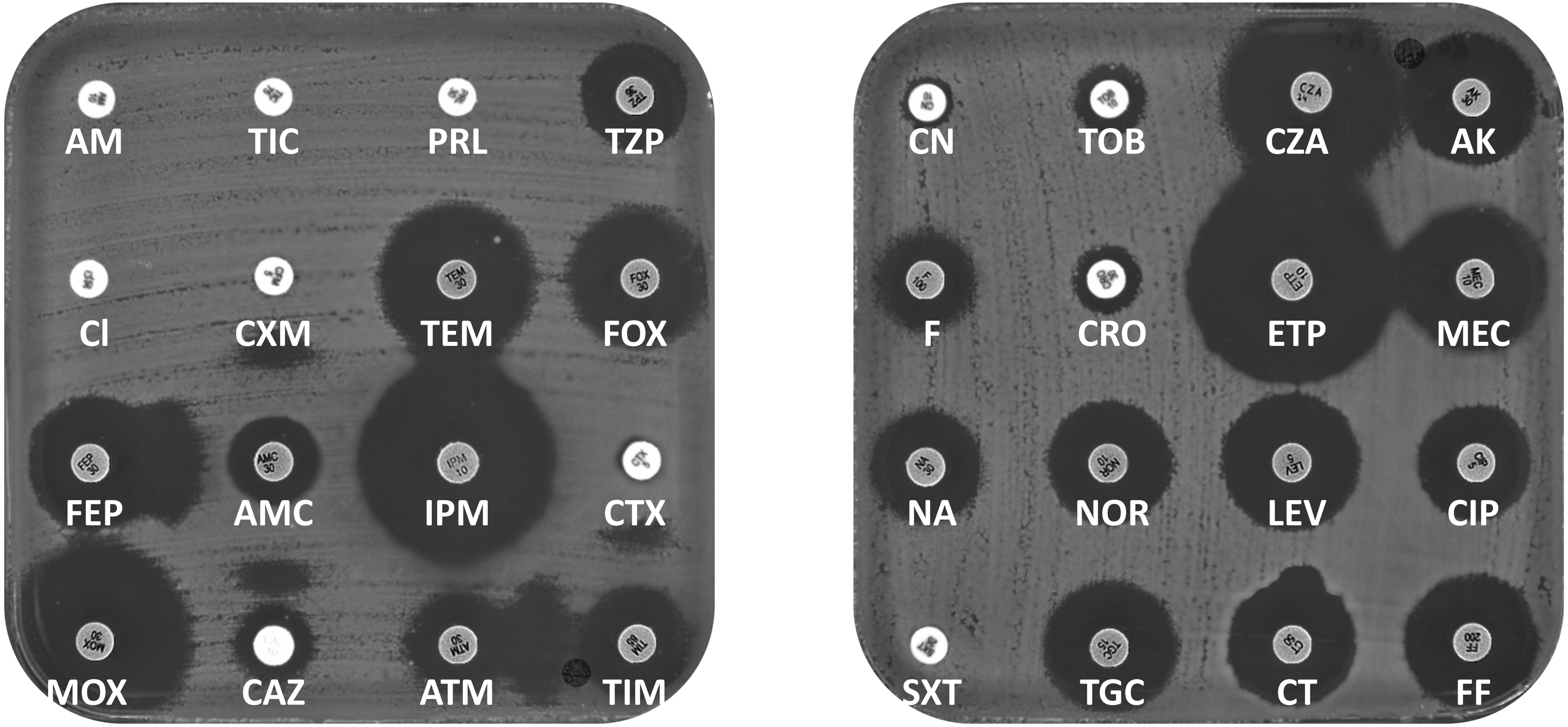

AM: amoxicillin, TIC: ticarcillin, PRL: piperacillin, TZP: piperacillin/tazobactam, CL: cephalothin, CFM, cefixime, TEM: temocillin, FOX: cefoxitin, FEP, cefepime, AMC: amoxicillin/clavulanate, IPM: imipenem, CTX: cefotaxime, MOX: moxalactam, CAZ: ceftazidime, ATM: aztreonam, TIM: ticarcillin/clavulanate, CN: gentamicin, TOB: tobramycin, CZA: ceftazidime/avibactam, AK: amikacin, F: nitrofurantoine, CRO: ceftriaxone, ETP: ertapenem, MEC: mecillinam, NA: nalidixic acid, NOR: norfloxacin, LEV: levofloxacin, CIP: ciprofloxacin, SXT: co-trimoxazole, TGC: tigecycline, CT: colistin, FF: Fosfomycin.
